# Stem Cell Proliferation and Quiescence—Two Sides of the Same Coin

**DOI:** 10.1371/journal.pcbi.1000447

**Published:** 2009-07-24

**Authors:** Ingmar Glauche, Kateri Moore, Lars Thielecke, Katrin Horn, Markus Loeffler, Ingo Roeder

**Affiliations:** 1 Institute for Medical Informatics, Statistics and Epidemiology, University of Leipzig, Leipzig, Germany; 2 Department of Gene and Cell Medicine, Mount Sinai School of Medicine, New York, New York, United States of America; 3 Department of Computing, Goldsmiths, University of London, London, United Kingdom; University of Auckland, New Zealand

## Abstract

The kinetics of label uptake and dilution in dividing stem cells, e.g., using Bromodeoxyuridine (BrdU) as a labeling substance, are a common way to assess the cellular turnover of all hematopoietic stem cells (HSCs) in vivo. The assumption that HSCs form a homogeneous population of cells which regularly undergo cell division has recently been challenged by new experimental results. For a consistent functional explanation of heterogeneity among HSCs, we propose a concept in which stem cells flexibly and reversibly adapt their cycling state according to systemic needs. Applying a mathematical model analysis, we demonstrate that different experimentally observed label dilution kinetics are consistently explained by the proposed model. The dynamically stabilized equilibrium between quiescent and activated cells leads to a biphasic label dilution kinetic in which an initial and pronounced decline of label retaining cells is attributed to faster turnover of activated cells, whereas a secondary, decelerated decline results from the slow turnover of quiescent cells. These results, which support our previous model prediction of a reversible activation/deactivation of HSCs, are also consistent with recent findings that use GFP-conjugated histones as a label instead of BrdU. Based on our findings we interpret HSC organization as an adaptive and regulated process in which the slow activation of quiescent cells and their possible return into quiescence after division are sufficient to explain the simultaneous occurrence of self-renewal and differentiation. Furthermore, we suggest an experimental strategy which is suited to demonstrate that the repopulation ability among the population of label retaining cells changes during the course of dilution.

## Author Summary

Hematopoietic stem cells (HSCs) are among the best-studied populations of adult stem cells. Commonly HSCs are considered to be in a so called *quiescent* state of reduced cellular turnover. However, it appears that even quiescent HSCs are activated into the cell cycle from time to time to support the continuous production of peripheral blood. Previous reports on cell kinetic studies using specific chromosomal labeling techniques suggest that all HSCs regularly undergo cell divisions on average once in two weeks. However, these results are challenged by recent experimental findings supporting the idea that HSC populations are heterogeneous with respect to their cell cycle activity. Applying an established model of HSC organization, we demonstrate that different data sets can be consistently explained by the assumption that HSCs are reversibly switching between a quiescent and an activated state. Based on this assumption, we provide a functional explanation for the experimentally observed biphasic label dilution kinetics of HSCs. We conclude that the ability of these cells to *reversibly* change between proliferation and quiescence is a critical and inherent property of the HSC system necessary for the sustained balance between stem cell self-renewal and the maintenance of functional blood cells.

## Introduction

The major task of hematopoietic stem cells (HSCs), beside the regeneration of the hematopoietic system after injury, is the lifelong supply of mature blood cells. There is consensus that even in the unperturbed, homeostatic situation there is the need for proliferation of HSCs in order to compensate the loss of cells due to differentiation. However, the turnover rate of HSCs is still controversially discussed [1]–[4]. Although a certain (low) proliferative activity of HSCs is accepted in general, it is still unclear whether quiescent HSCs are regularly activated into cell cycle and to what extent each cell contributes to hematopoiesis over the life time of an organism. This controversy directly relates to the understanding of proliferation-related phenomena such as stem cell exhaustion and aging as well as the protection of genome integrity in order to circumvent the development of leukemic malignancies, originating from the HSC compartment.

The most accepted assay to determine the “quality” of HSCs is the transplantation of these cells into lethally irradiated animals. The existence of “true” HSCs among the transplanted cells is generally affirmed if the cells engraft in the bone marrow (or other blood producing tissues), reestablish normal hematopoiesis, and rescue the animal. There is accumulating evidence, that the repopulation ability of HSCs is directly linked to their proliferative activity. In particular it has been reasoned, that high repopulation potential of HSCs is associated with proliferative quiescence [5]–[7]. This suggests a protective mechanism of stem cell quiescence which is commonly associated with the action of hematopoietic niches [1]. Still, it is unclear how the control of stem cell quiescence is maintained while at the same time the contribution to the production of peripheral blood cells is facilitated.

A common method to investigate the cell kinetics of HSCs is DNA labeling using e.g. Bromodeoxyuridine (BrdU) [8]. BrdU is a thymidine analogue that is incorporated into newly synthesized DNA during cell division and can be detected using antibody staining. With this method it has been demonstrated that the actual proportion of actively proliferating HSCs (i.e. the proportion of cells in S -phase) in a homeostatic system is only about 5% at any given time point. Additionally, it could be demonstrated that at the same time almost all HSCs can be labeled within a period of 3 to 6 months, demonstrating the turnover of the whole stem cell pool [9]. These findings were complemented recently by a similar study using a more sophisticated protocol for the enrichment of HSCs [3]. The authors of the latter study conclude from the data of both experiments that all HSCs divide regularly with a small but common turnover rate and that the kinetics of BrdU label uptake and dilution show exponential behavior.

However, it has been recently demonstrated in two independent studies that the simple explanation of a common turnover rate of all HSCs does not hold [4],[10]. The observed label dilution of either BrdU [4] or an equally suited GFP-histone label [4],[10] suggests a biphasic decline kinetic in which a subpopulation of more rapidly dividing cells is responsible for an accelerated early decline, whereas a slowly dividing subpopulation accounts for the decelerated dilution on longer time scales. Furthermore, Wilson et al. [4] show that the kinetics of label uptake are much faster as compared to the label dilution, suggesting that the administration of BrdU during the labeling process itself perturbs homeostasis in the stem cell compartment.

The authors of both studies argue that the observed label dilution can only consistently be described in the context of a two population model. Using similar mathematical modeling approaches that assume distinct subpopulations of HSCs, those authors are able to quantitatively describe the observed biphasic label dilution kinetics.

Although measurements of the fraction of label retaining cells within purified stem cell populations provide important information for the understanding of the regulation of cellular turnover, they do not provide conclusive insights about the repopulation potential of the cells on their own. The “gold standard” for accessing the repopulation potential of stem cells is the use of repopulation assays. Label retention experiments using BrdU are insufficient in this respect, since the detection of incorporated label requires the fixation of the cells and excludes their use in repopulation assays. An attractive alternative is histone-GFP fusion protein already applied in different tissues [11],[12]. Other versions have been recently developed and applied in the context of HSC kinetic studies [4],[10].

A number of mathematical models have been developed to explain experimental results on label uptake and dilution kinetics in various stem cell systems [3],[4],[13]. Most of these models use ordinary differential equations to describe the average label content of a homogeneous population of cells that regularly undergo cell division. Considering further levels of heterogeneity, this population is divided into a hierarchy of distinct subpopulations with different (cell kinetic) parameters. However, a strict compartmentalization of HSCs in the context of a unidirectional differentiation hierarchy does not provide a mechanistic explanation of the cellular interaction within the HSC population nor for the interactions with microenvironmental cues. In particular, these models fail to account for clonal differences among individual HSCs and for the assessment of the repopulation potential of individual (selected) cells. Therefore, we argue that for the analysis of label kinetics in this broader context of HSC organization the simplified representation by compartment models has to be complemented by a description on the individual cell level that includes aspects of cell-cell and cell-environment interaction.

Over the last years we have developed and improved an appropriate mathematical model of HSC organization which fulfils these criteria and which has been successfully applied to explain a wide range of diverse phenomena such as clonal repopulation, individual cell fates decisions, lineage specification or leukemia development and treatment effects [14]–[18]. With respect to cell kinetic control, our model considers two distinguishable functional states, namely quiescence and proliferation, and HSCs are assumed to be able to reversibly change between these states. Application of the model to the situation of label dilution offers a mechanistic interpretation of the biphasic decline in the context of an adaptive, self-organized stem cell population. Most importantly, our model explains how label dilution influences the composition of the stem cell compartment over time and it implies a strategy to experimentally demonstrate the predicted temporal changes in the composition of HSC populations with respect to their repopulation potential.

## Results

### Label dilution in the context of compartment models

**Figure 1:**
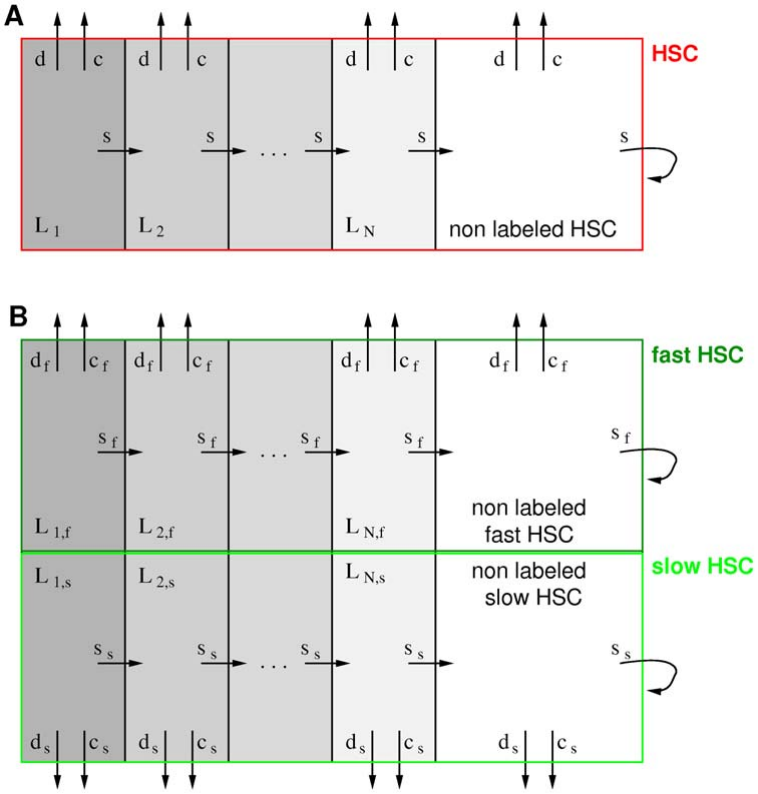
Compartment models of stem cell organization. **A.** The red boxed area indicates the population of HSCs. Each cell within this population undergoes cell division with rate *s* (generating two daughter cells), differentiation with rate *d* and cell death with rate *c*, shown by the arrows. The processes of differentiation and cell death lead to the removal of the cell from the HSC compartment. Upon label administration a certain fraction *F_0_* of HSCs gets initially labeled. As *N* subsequent divisions are necessary to dilute the label below the detection threshold, this can be visualized by a sequence of N compartments named L_1_ to L_N_, shown in grey. Cells within theses boxes undergo cell division (transit from L_i_ to L_i+1_), differentiation, and cell death with the same rates as non-labeled cells. After the *N*th division the cells are no longer distinguishable from unlabeled HSCs. **B.** The population of HSCs is composed of two, subpopulations, indicated by the lower (light green) and upper (dark green) boxes, which differ in their specific rates for cell division (*s_f_* and *s_s_*), differentiation (*d_f_* and *d_s_*) and cell death (*c_f_* and *c_s_*). Otherwise, the fast and the slow dividing subpopulations behave identical to the case illustrated in subfigure A. Labeled cells are present in both these subpopulations and need to undergo *N* subsequent divisions to dilute label below the detection threshold.

We have re-analyzed BrdU label dilution data published by Kiel et al. [3] and Wilson et al. [4] using different mathematical approaches, that employ population based compartment models and single cell-based stochastic modeling. Kinetics of label uptake are not considered as they most likely do not reflect the homeostatic situation [4]. The simplest compartment model to explain the label dilution process assumes a homogeneous population of stem cells which regularly undergo divisions with an average turnover rate *s*. As the overall number of stem cells in the compartment needs to be constant for the assumed homeostatic situation, cell amplification with rate *s* needs to be balanced by the processes of immediate differentiation (with rate *d*) and/or cell death (with rate *c*). Please note that the latter two processes also lead to a loss of label-retaining HSCs and are, together with cell division, denoted as diluting events. Starting with a fraction *F_0_* of labeled cells it is assumed that HSCs need to undergo a fixed number (*N*) of cell divisions to dilute the label below the detection threshold. This process of label dilution is represented by *N* sub-compartments (denoted as L_1_ to L_N_) within the HSC population representing the different labeling states. The model layout is sketched in Figure 1A.

**Figure 2:**
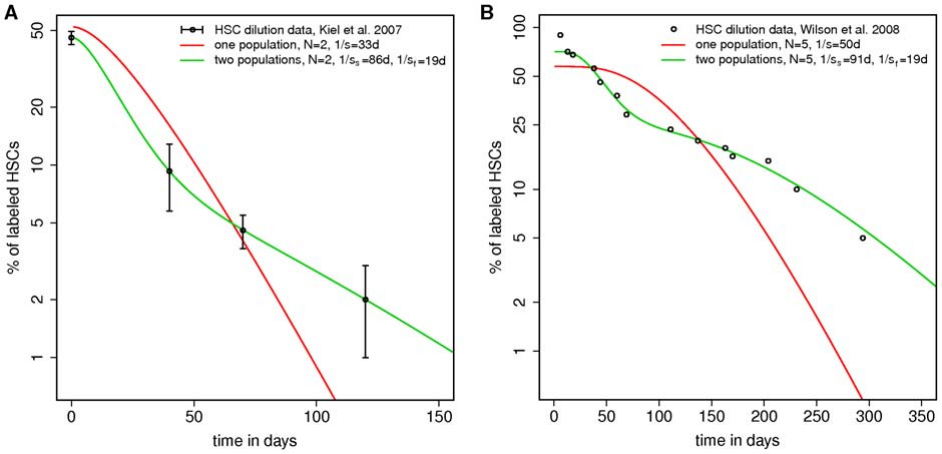
Kinetics of label dilution described in the context of compartment models. **A.** The red and the green curves represent best fit scenarios for the data on BrdU label dilution obtained from Kiel et al. (black circles, mean+/−SD) [3]. The red curve corresponds to the one compartment model (compare Figure 1A) in which *N* = 2 divisions are necessary to dilute the label below the detection threshold. The green curve corresponds to the two compartment model (cf. Figure 1B) with parameters 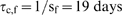 and 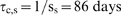, also assuming *N* = 2 divisions until label dilution. **B.** The same compartment models are fitted to the data on BrdU label dilution obtained from Wilson et al. (black circles, mean values) [4], assuming that *N* = 5 divisions are necessary for label dilution. Although the one compartment model fails to describe the data (red curve), the two compartment model (green curve) captures the overall behavior for 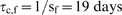 and 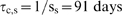.

Assuming that the occurrence of division, differentiation, and cell death can be described by a Poisson process (i.e., individual events occur with a low but constant rate), it follows that the time to the next dilution event (either division, differentiation or cell death) is exponentially distributed with characteristic rate 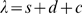, in which 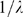 is proportional to the characteristic time until the next event [19]. However, if more than one division event is necessary to dilute the BrdU label below the particular detection threshold, the time to the occurrence of the *N*th dilution event is no longer exponentially distributed, but follows a gamma distribution with parameters (*N*, 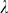) (see e.g. [20]). Taking the parameters from the relevant publications, that means BrdU positive cells become BrdU negative (i.e., undetectable) after *N*≈2 (Kiel et al. [3]) and *N*≈5 (Wilson et al.[4]) divisions, respectively, and applying a corresponding Poisson model to the data, the assumption of a homogeneous population fails. The red curve in Figures 2A and B corresponds to the best fit scenario of the corresponding one compartment Poisson models and illustrates the disagreement of this simple model with the experimental data.

Within a more elaborated model one assumes that the HSC population is composed of two independent subpopulations with identical structure but different rates of division, differentiation and cell death. In this interpretation, the HSC population consists of a fraction of fast dividing cells (with characteristic rate 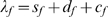) and a fraction of slow dividing cells (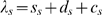). A sketch of the model is shown in Figure 1B.

In the context of such a model both available data sets can be described consistently (green curves in Figure 2A and B). Assuming that BrdU+ cells become undetectable after *N* = 2 (Kiel et al.) and *N* = 5 divisions (Wilson et al.), respectively, the estimated characteristic rates 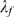 for the fast dividing subpopulation and 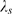 for the slowly dividing subpopulation are almost identical for the different data sets. The average turnover time (defined as the average time of an individual cell until the next division event, given as 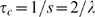) are estimated as 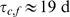 for the fast dividing subpopulation and 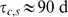 for the slowly dividing subpopulation.

Similar two-population models of HSCs have also been discussed by Wilson et al. [4] and Foudi et al. [10]. Whereas, Foudi et al. describe the dilution of GFP histone label as the superposition of two strictly distinct HSC subpopulations, Wilson et al. assume that differentiating cells among the slowly dividing HSCs transit into the fast dividing HSC population instead of leaving the HSC compartment directly. This additional, uni-directional flux between the two HSC sub-populations leads to lower estimates for the characteristic rates 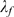 and 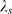 compared to the model with two independent HSC subpopulations.

The ability to consistently describe both data sets, the one published by Kiel et al. [3] as well as the one published by Wilson et al. [4] in the context of a common model (in which only the number of divisions to undetectability differs), is a strong argument in favor of an inherent heterogeneity among HSCs. As outlined above, there is evidence to assume that the slower turnover corresponds to a population of largely quiescent HSCs which are only activated into cell cycle on long time scales [1],[4],[14]. In contrast, the fast turnover represents those HSCs that are actively proliferating. The resulting overall kinetics of label dilution appears as a superposition of the fast and the slow kinetics.

However, population-based models that assume a strict distinction between fast and slow dividing HSC subpopulations cannot provide a functional explanation on how these two facets of HSCs can be confined in a unified picture. Especially the mutual regulation between the compartments and their response to changing environmental conditions is not appropriately reflected in these representations. Moreover, in the context of fixed constants for proliferation, differentiation and cell death these models fail to account for phenomena of repopulation after HSC depletion or transplantation.

### Label dilution in the context of a mechanistic model of HSC organization

**Figure 3:**
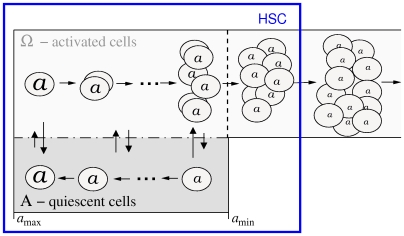
Modeling concept of a self-organized HSC population. **A.** The model setup is characterized by two different signal contexts (A and 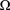). Cells can reversibly change between A and 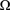 depending on the cell numbers and the cell specific affinity *a*. Whereas activated cells in 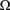 undergo divisions and exponentially degrade their cell specific affinity *a*, cells in A are quiescent and preserve/regain their affinity *a*. Further details of the model are given in [14],[17] and in the Supporting Information Text S1. The blue box indicates the region in which cells are considered as HSCs according to a certain purification procedure.

Alternatively, we propose a different view in which the strict compartmentalization into a fast and a slow dividing HSC subpopulation is replaced by the ability of individual HSCs to adapt their cycling status in response to environmental signals, namely, to change reversibly between cellular quiescence and proliferation [14],[17]. For the quantification of our concept we developed a single cell-based model assuming that HSCs reside in either of two *signaling contexts*, named A and 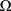, which impose different effects on the cellular development. In particular, context A is inspired by the concept of a stem cell supporting niche and promotes cellular quiescence and regeneration. In contrast, context 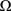 represents an escape of HSCs from the niche-signals, and promotes proliferation and differentiation. A cell's tendency to switch from one context into the other is determined by the cell number in the target context (i.e. the “packing density”) and by a cell specific affinity *a* to reside in context A. Because residence in context A is necessary to prevent differentiation and, therefore, implicitly to maintain the HSC population, the variable *a* can be interpreted as a measure of the repopulation potential of individual cells. As the cell specific affinity *a* is gradually lost in context 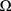 but regained in A, the system is able to establish a dynamically stabilized equilibrium, balancing quiescent cells in A and proliferating cells in 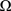. If the cell specific affinity *a* drops below a certain threshold *a*_min_, the cells have lost the ability to changing back to context A, and are committed to undergo further proliferation and differentiation. A sketch of the model is provided in Figure 3. In our model the population of HSCs (blue box in Figure 3) is represented by a mixture of all quiescent cells in signaling context A and a fraction of activated cells in 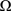. The fraction of activated cells can be used to model different cell sorting efficiencies, i.e. populations with varying proportions of long-term repopulating cells. See also Material and Methods for further details of the model.

As previously suggested this model is well suited to explore the kinetics of label dilution [14]. To do so, each cell is additionally characterized by a variable *b* (*b* in [0,1]) describing the current label content. We make the simplifying assumption that upon division the two daughter cells retain half of the parental label content. Although the label is segregated with individual chromosomes our assumption does well describe the average case. In order to adapt the simulation model to the available data on BrdU label retention, we adjust parameters that describe the activation of quiescent cells from A into 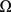 (regulating the residence times in A and thus the turnover of the quiescent cells) as well as the cell cycle times of the activated cells in 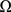 (corresponding to the turnover of the activated cells).

To simulate label dilution we start from a homeostatic system. At time point *t* = 0 a certain fraction *F_0_* of cells is labeled with initial label content *b_0_* (without loss of generality we use *b_0_* = 0.5 for all labeled cells). For the data by Kiel et al. [3] the fraction of labeled cells is best estimated as *F_0_* = 45% which is close to the measured fraction of labeled cells after the initial uptake. For the data by Wilson et al. [4] the fraction is adjusted to *F_0_* = 71% which is closer to the fraction of label retaining cells at *t* = 10 d after stop of label administration. This time point has also been chosen as the initial point of our model analysis, because Wilson et al. report that the initial dilution phase is potentially biased by cytotoxic effects of the BrdU label [4]. Besides the initial fraction of labeled cells *F_0_*, our simulations of the two data sets differ only in the detection threshold of the label. For the data by Kiel et al. [3] the threshold is set to *b_t_* = 0.2, whereas for the data by Wilson et al. [4] it is set to *b_t_* = 0.02 (reflecting dilution after *N* = 2 and *N* = 5 divisions, assuming *b_0_* = 0.5, respectively).

**Figure 4:**
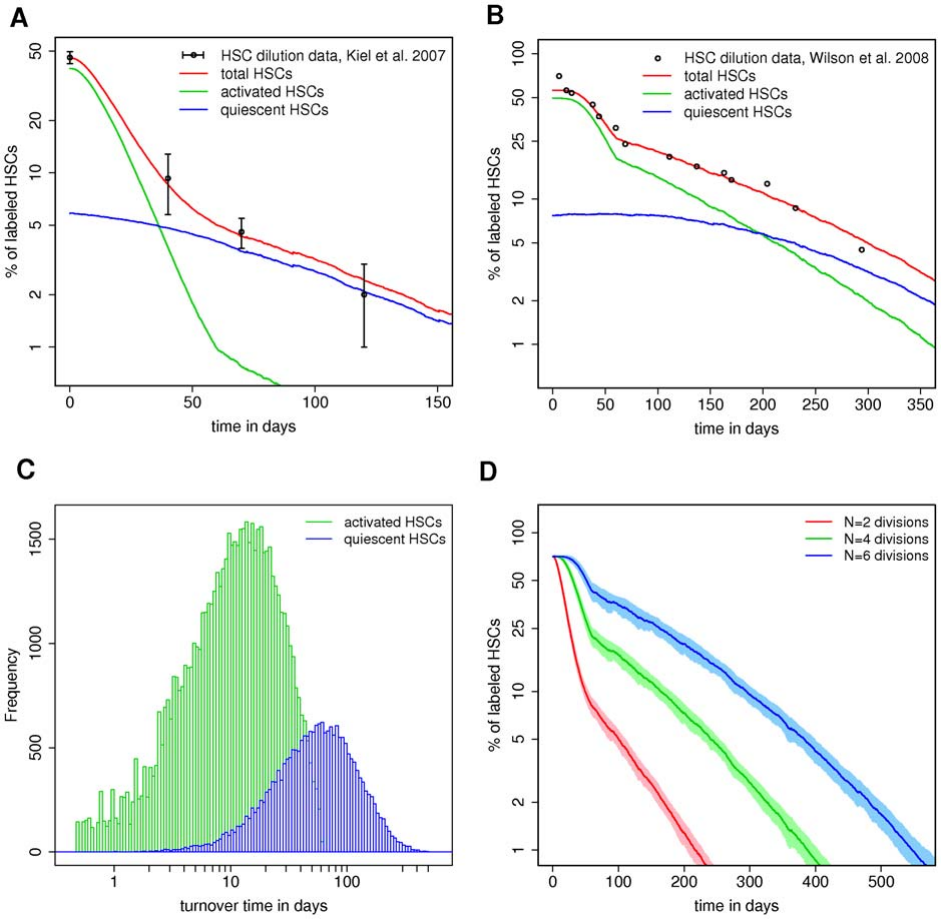
Kinetics of label dilution in the context of the single-cell based model. **A.** Optimal fit of the single cell-based model (red curve, average of 100 simulation runs) to the particular data by Kiel et al. [3] (black dots +/−SD). The corresponding green and blue curves show the corresponding fraction of activated and quiescent cells among the label retaining cells, respectively. *F_0_* = 45% of HSCs are initially labeled, *N* = 2 divisions are necessary to dilute the label below the detection threshold. **B.** Corresponding fit for the data by Wilson et al. [4]. *F_0_* = 71% of HSCs are initially labeled, *N* = 5 divisions are necessary for label dilution. **C.** Distribution of individual turnover times in the simulation for activated (green) and quiescent cells (blue). **D.** Percentage of label retaining cells as a function of time, depending on the number of divisions *N* to dilute the label. Dark lines are the average values over 100 simulations, shaded regions indicate +−SD.

As indicated in Figure 4 the model describes both experimental situations without the need of any additional assumptions. The simulations demonstrate that a model, in which the subpopulations of fast and slowly dividing cells are not fixed, but in which there is an ongoing traffic between cellular quiescence and proliferation, consistently reproduces the characteristic biphasic decline of label retaining cells. The best fit of the simulation results and the experimental data is achieved by choosing the average turnover time of the activated cells 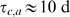 and of the quiescent cells 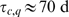. As the individual values of the turnover times show a high variability, we provide their distributions in Figure 4C. It can be seen that there is a significant distinction between the two dynamical regimes with a major contribution of the quiescent stem cells to extremely long turnover times (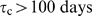). The difference of the average turnover times estimated from our single cell-based model as compared to the above stated compartment models is a result of the conceptually different explanations.

The comparative study of two data sets with different thresholds for the detection of label retention already indicates that there is a correlation between the fraction of label retaining cells and the available measurement procedure. However, besides the detection threshold *b_t_* also the initial label content *b_0_* (generated during label administration) matters. In this sense, the number of divisions *N* needed to dilute the initial label content *b_0_* below the detection threshold *b_t_* is the critical parameter. For the situation that *F_0_* = 71% of the cells are initially labeled with homogenous content *b_0_* we show in Figure 4D the fraction of label retaining as a function of time given that *N* = 2, 4, 6 divisions are necessary to dilute the label. Whereas for *N* = 2 almost no label retaining cells are detectable after one year, more than 5% of such cells are detected at the same time point using a more sensitive detection method (*N* = 6).

It is an idealization that all cells have the same initial label *b_0_* after a period of label administration. In fact, the initial label content of individual cells might greatly vary depending on the number of divisions in the presence of a labeling substance and the efficiency of label uptake itself. Within the single cell-based model framework we can directly study, how the distribution of the label content after label administration influences the kinetics of dilution. Applying a moderate distribution of initial values *b*_0_ and keeping the mean level of label content constant we can show that the initial distribution has only a minor effect on the dilution kinetics (see Supporting Information Text S1).

### Prediction of repopulation dynamics

Application of the single cell-based model allows for each individual cell to access its current cycling status, its label content, and its divisional history for every time point during label dilution. In this sense we are able to follow explicitly the composition of the population of label retaining cells over time. As it is illustrated in Figure 4A and B, the overall kinetics of label dilution (shown in red) represent a superposition of the label dilution of the quiescent (blue line) and the activated cells (green line). Again, it should be emphasized that these are not considered as independent populations. Instead, all stem cells can reversibly transit between the two different activation states. Actual loss of label content in HSCs (due to cell division) only occurs if the cells are in the activated status, mediated by the signaling context 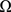. However, as also quiescent cells in context A can become activated, divide in context 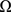, and potentially reenter the quiescent state, dilution is also expected among slowly dividing (quiescent) HSCs.

In the early phase of dilution, the label retaining cells contain a significant number of activated cells which originate from the initial labeling routine. The fraction of activated cells decreases rather quickly since these cells undergo division on average every 10 days if they do not change into quiescence (cf. Figures 4A and B). However, even on longer time scales, it is possible to detect activated HSCs among the label retaining cells. These cells almost exclusively derive from the occasional activation of previously quiescent stem cells. Consequently, due to the slow turnover of the quiescent HSCs, their fraction increases among the label retaining cells. In this sense, the composition of the pool of label retaining cells changes over time and our model analysis suggests that the population of label retaining cells detected in late phases of dilution exhibit on average a higher repopulation potential compared to the label retaining cells isolated in the early dilution phase. For a quantitative validation of this prediction one needs to perform competitive retransplantation experiments at varying time points. As mentioned before, in such experiments BrdU needs to be replaced by an alternative labeling technique such as GFP histone labeling.

In order to compare the repopulation ability of label retaining (L+) and non-label retaining (L−) HSCs over time, we run multiple simulations in which initially (and without loss of generality) *F_0_*≈71% of HSCs in an homeostatic system are randomly labeled. At different time points during label dilution, the entire population of HSCs is separated into L+ and L− cells (according to the detection threshold *b_t_* = 0.02). From each of these populations, at each time point, 20 randomly chosen cells are transferred into an empty model system, mimicking the situation of in vivo repopulation assays. In order to account for competitor cells, we “co-transplanted” 20 HSCs randomly selected from a homeostatic system in which no labeling routine had been applied. These cell numbers roughly correspond to a transplantation regime in which 1000 Lin- Sca1+ c-Kit+ cells (containing about 2% ≈20 stem cells [21]) are co-transplanted with 1×10^6^ unselected bone marrow cells (also containing ≈20 stem cells [22],[23]). As the number of transplanted cells is well below the number of HSCs in the homeostatic situation the system expands and establishes equilibrium between cells from the L+ (or L−) donor population and the population of competitor cells. The engraftment level (i.e., the fraction of cells derived from the donor population, usually detected by discriminating surface markers, such as CD45.1 vs CD45.2) is commonly used as a measure for the quality of the transplanted cells.

**Figure 5:**
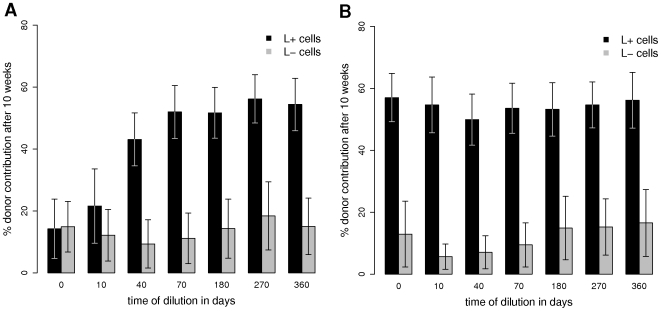
Engraftment levels as a function of time of dilution. **A.** The average level of donor engraftment for the L+ (black) and L− cells (grey) is shown as a function of the time of dilution. Initially *F_0_* = 71% of all HSCs are randomly labeled. At each time point of dilution 20 randomly chosen L+ or L− cells are transplanted competitively with 20 randomly selected HSC from the host system. Engraftment levels of the donor cells are assessed for the time point 10 weeks after transplantation. Simulation results are averages of 100 independent realizations +/−SD. **B.** Identical setup to subfigure A, only the labeling routine is restricted to the most primitive cells (*F_0_* = 100% for cells with cell specific affinity *a*>0.9).

Predicted engraftment levels 10 weeks after transplantation for the L+ and L− subpopulations are shown in Figures 5A. The x-axis indicates the time during dilution at which the L+/L− cells had been isolated form the model system. The pronounced initial increase of the repopulation potential of L+ cells corresponds to the above prediction that during early dilution the population of L+ cells contains many initially labeled, activated cells. While the fraction of activated label retaining cells decreases as these cells divide, the corresponding fraction of label retaining, quiescent cell increases proportionally. This leads to the overall increase of repopulation potential of the L+ cells. Nevertheless, even the predicted rare activation of quiescent HSCs into cell cycle leads to a final exhaustion of L+ cells on long time scales (cf. Figure 4D).

Although competitive retransplantation experiments of L+ and L− are only reported for cells isolated at one particular time point during dilution in the relevant studies of Wilson et al. [4] (transplants of cells isolated at day 213 of label dilution) and Foudi et al. [10] (transplants of cells isolated at day 140 of label dilution), the results are comparable to our simulation results (day 180, Figure 5A). The experimental results indicate engraftment levels of the L− population around 20%, which is in good agreement to the predicted engraftment levels of the simulation model. The engraftment levels for the L+ population of around 80% are slightly underestimated by our model. Nevertheless, the general difference between the two populations in their engraftment level is well reflected and our predictions on the changes in the engraftment level of the L+ cells over time should be even more pronounced in the experimental situation.

In order to illustrate that the changes in the repopulation potential (i.e. achievable level of donor engraftment) of the L+ HSCs are attributed to the initial configuration of the labeled cells before dilution, we study another contrasting situation. Now, the initial label incorporation is not uniformly distributed among activated and quiescent HSCs, but largely restricted to the most primitive cells which are almost completely labeled (*F_0_* = 100% for cells with cell specific affinity *a*>0.9). This primitive population comprises a significant amount of quiescent HSCs but is poor in activated cells. Therefore, we predict that an initial increase of the repopulation potential among the L+ cells does not occur as a dilution of the fraction of activated label retaining cells is negligible. Indeed, the corresponding simulations in Figures 5B quantify this prediction. The engraftment levels of L+ cells isolated in early dilution phase do not differ from the levels at later stages.

Although the repopulation potential among the L− cells remains almost constant in the first situation (i.e., activated and quiescent cells are equally labeled, cf. Figure 5A), a slight increase is predicted for the second situation (i.e., preferential labeling of quiescent cells, cf. Figure 5B). In the latter case, the L− cells in the early dilution phase are almost exclusively activated HSCs without a significant amount of quiescent cells among them. However, in the course of dilution, the quiescent cells that have once been activated are successively replaced by cells with reduced label content due to their divisional activity. This increase of the fraction of quiescent L− cells results in a final increase of the overall repopulation potential among the L− HSC. An experimental validation of this prediction would strongly support the hypothesis that activated stem cells, which have lost their label due to cell division, can reversibly switch back into quiescence.

## Discussion

The biphasic decline of the fraction of label retaining cells during in vivo label dilution is a strong argument in favor of heterogeneity among HSCs. For a functional explanation of the observed heterogeneity, we propose a concept in which each HSC can either be activated into cell cycle or stay in a quiescent state. Using a corresponding single cell based mathematical model of HSC organization we demonstrate that these assumptions are fully consistent with different sets of previously published data on BrdU label dilution.

Additionally, we confirm that the data on BrdU label dilution can in principle be explained in the context of simpler compartment models in which at least two distinct subpopulations of HSCs are considered with different, fixed turnover rates. However, these population-based approaches are not suited to study clonal effects and competitive retransplantation experiments. It is the advantage of our model not only to describe the differential dynamics in two distinct subpopulations but to provide a mechanistic understanding on how the dynamic equilibrium between these two states is maintained. Based on this cellular perspective the resulting, overall kinetics of label dilution are assessed in a biologically meaningful context.

The idea that HSCs appear as an inseparable, heterogeneous mixture of cells containing quiescent and activated cell populations is long-standing [15],[24],[25] and contradicts the theory of clonal succession which explains differentiation by a sequential and irreversible activation of quiescent HSCs (see e.g. [26]–[28]). However, this idea of a heterogeneous population of HSCs is still underappreciated compared to the prevailing view that HSC development occurs as a unidirectional transition of cells through distinct and separable subpopulations with declining repopulation potential. Appropriate quantitative models that account for the adaptive regulation of HSC numbers by the dynamic regulation of stem cell activation and quiescence including the occurrence of reversible developments have already been established and verified for a broad range of phenomena [14],[18],[29],[30]. Whether such models are formulated in terms of partial differential equations or using a more intuitive single cell based model as the one introduced here depends on the particular scientific question and on the available resources (see e.g. [31],[32]).

Our model approach suggests that residual levels of label retention in the unperturbed situation, especially on long time scales, are the most reliable measure to determine the turnover times within the quiescent cell population. However, we could also show that the fraction of label retaining cells is highly dependent on the particular experimental threshold for the label detection in individual cells. Furthermore, our model supports the idea that division-dependent label retention after long chase periods is a suitable means for the enrichment of long term repopulating stem cells. In contrast to the proposed compartment models, the general class of adaptive models it suited to quantitatively study and explain the effects of transient activation of HSCs using cytokines (such as G-CSF or 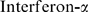) or cytotoxic drugs (e.g. 5FU).

We have previously shown that our model setup proved useful to model competitive retransplantation assays in various experimental settings [16],[33]. Applying the model to the situation of label dilution kinetics we simulate transplantation experiments for different time points during chase and provide a quantitative understanding of the changes in engraftment levels. In particular, we demonstrated that the slow label dilution among the quiescent cells and the fast dilution among the activated cells lead to increasing engraftment levels of the L+ population over time. In this respect, the changes in the repopulation ability of L+ and L− cells directly reflect the changes in the underlying composition of the transplanted cell populations, i.e. the fractions of activated vs. quiescent cells, rather than a change of the properties of the individual cells.

Quiescence of HSCs is regularly associated with the affiliation to hematopoietic niches [2],[34]. These particular, spatial environments exert a protective action in which HSCs are held in a rather inactive state while they maintain their full repopulation ability. This perception fits well with our modeling approach in which the concept of quiescence is primarily motivated by the action of the hematopoietic niches to which HSCs can reversibly bind. From a conceptual point of view, and substantiated by our simulation results, we argue that proliferation and quiescence are just two sides of the same “stem cell coin”. It is precisely the volatile interplay between these two facets that facilitates the simultaneous occurrence of HSC maintenance and differentiation in a dynamically stabilized system. This implies that the dualism in the appearance of HSCs (activated vs. quiescent) is an inherent system property. This dualism and the reversibility of the actual cell state make it highly questionable to consider these populations as being independent from each other.

## Materials and Methods

### Data sources

Data on BrdU label dilution has been obtained from [3], Figure 3C. The relevant HSC population has been sorted using flow cytometry according to the surface marker combination CD150^+^, CD48^−^, CD41^−^, lineage^−^, Sca-1^+^, c-Kit^+^. A second, more extensive data set was extracted from [4], Figure 2E in which a similar population of HSCs, defined as CD150^+^, CD48^−^, CD34^−^, CD135^−^, lineage^−^, Sca-1^+^, c-Kit^+^, has been used to study the dilution of BrdU label.

### Compartment models for label dilution

The dynamics (i.e. the changes in cell population size) of a homogeneous population of HSCs are characterized by the rates for the occurrence of cell division *s* (i.e. one cell divides into two), differentiation *d* (i.e. loss of the HSC specific characteristics) and cell death *c* (i.e. immediate loss of the cell). As the size of the population needs to be constant in an unperturbed, homeostatic situation the rates of division on one side and of differentiation and cell death on the other side need to be equal, thus *s = d+c*.

Label retention of an initial fraction *F_0_* of HSCs that need to undergo *N* subsequent divisions until dilution is conveniently represented by a sequence of *N* compartments named L_1_ to L_N_ (compare Figure 1). Upon division within compartment L_i_ the daughter cells lose label content; in particular they retain half the parental label. This is modeled by the transit into compartment L_i+1_. As the rate constants for division, differentiation and cell death are the same as for the unlabeled population, the fraction of cells in compartment L_i_ is described by an ordinary differential equation [13]of the form:
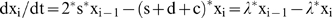
in which 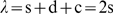 (the latter equality is implied by the constraint of an homeostatic system, see above) is the characteristic rate for the occurrence of either division, differentiation or cell death. For compartment L_1_ the first part (2*s*x_0_) vanishes since there is no influx of cells with higher label content (i.e; x_0_ = 0).

The time until a cell has passed through a sequence of *N* such identical compartments is described by gamma distribution with parameters *N* and 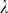, given that the individual events can be described by a Poisson process [20]. Therefore, the fraction of cells within compartments L_1_ to L_N_ is given as 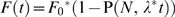 in which 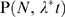 is a gamma distribution (also denoted as an incomplete gamma function, see [35]).

Assuming that HSCs consist of two distinct subpopulations with different characteristic rates 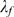 and 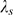 instead of one such population, the fraction of labeled cells in compartments L_1,*f*_ to L_N,*f*_ and L_1,*s*_ to L_N,*s*_ is given as 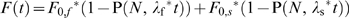. The fractions *F_0,f_* and *F_0,s_* correspond to the fraction of cells that are initially labeled among the fast and the slow dividing subpopulation. Under the assumption that label uptake occurs with equal probability within the two populations, the ratio *F_0,f_*/*F_0,s_* is a measure of overall ratio of fast and the slow dividing HSCs.

The curves in Figure 2 are obtained by fitting the one and the two-compartment model to the available data sets. Fits are obtained using the least-square fitting routine of the software *gnuplot*. Best fit parameters are: Figure 2A (data from [3]), *N* = 2, one compartment model: *F_0_* = 0.57, 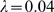, two compartment model: *F_0,f_* = 0.46, 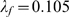, *F_0,s_* = 0.25, 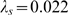; Figure 2B (data from [4]), *N* = 5, one compartment model: *F_0_* = 0.52, 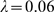, two compartment model: *F_0,f_* = 0.37, 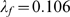, *F_0,s_* = 0.09, 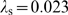.

### Model of adaptive HSC organization

Our simulation model of HSC organization is implemented as an agent-based model [36] with discrete time steps in which each individual cell is described as an independent agent. The state of each cell (i.e. its affinity *a*, the residence in signaling context A or 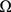, and its position in cell cycle) is updated according to a set of specified rules that include a number of stochastic elements (e.g. for the transitions between the signaling contexts). Details of the implementation as well as a list of used parameters are provided in the Supporting Information Text S1.

In contrast to former versions of the model, which used a fixed G1 phase of the cell cycle for cells in signaling context 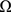 [14],[17], we assume that the duration of G1 phase for these cells is exponentially distributed resulting in an average turnover time 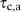. The residence times of cells in context A are characterized by the average turnover time of the quiescent cells 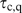. This, in turn, is related to the probability for changes between context A and 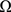 (and vice versa) which are dynamically regulated and depend in particular on the cell numbers in the target context and on the cell specific affinity *a*.

### Model representation of BrdU label

In the model, BrdU label content of each individual cell is represented by a variable *b*. In the daughter cells, *b* decreases in the dilution situation as *b*_daughter_ = 0.5*b_parent_ (i.e., asymptotically approaching *b* = 0 for extended dilution periods).

To account for the initial configuration of labeled cells after label administration we assume, that a fraction *F_0_* of all cells contain a certain amount of label *b = b_0_*. The detection threshold *b_t_* is chosen such that *N* divisions are necessary to dilute the initial label *b_0_* below the threshold value (e.g. for *b_t_ = 0.2* a cell with *b_0_ = 0.5* has to undergo *N = 2* division to become undetectable).

Under the simplest assumptions *b_0_* is identical for all labeled cells. However, to study the influence of the initial label distribution, we used an alternative scenario in which *b_0_* for each individual cell was chosen according to beta distribution with varying parameters (see Supporting Information Text S1).

The model description of labeling and dilution also applies to suitable alternative labeling methods besides BrdU such as GFP-conjugated histones.

## Supporting Information

Text S1 Supporting Information.
(0.28 MB PDF)
