# Supplementary material for: Stem Cell Proliferation and Quiescence—Two Sides of the Same Coin
Source: PLoS Comput Biol. 2009 Jul 24;5(7):e1000447. doi: 10.1371/journal.pcbi.1000447 (PMC2704962; doi:10.1371/journal.pcbi.1000447)
Supplement: Text S1 — Supporting Information. (0.28 MB PDF) [file pcbi.1000447.s001.pdf]

Stem Cell Proliferation and Quiescence - Two Sides of the Same Coin - Supporting Information Text S1

Ingmar Glauche, Kateri Moore, Lars Thielecke, Katrin Horn, Markus Loeffler, Ingo Roeder

1 Simulation algorithm and model equations

The described model of HSC organization is mathematically represented as a single-cell based, stochastic process. I.e., the development of each individual cell in the system is simulated according to a set of defined rules including stochastic decisions. These rules are applied at discrete time steps (Δt =1 hour) to simultaneously update the status of all model cells.
The status of each model cell is given by its cell specific affinity a, its membership to a signaling context $m \in \{A, \Omega\}$, and its position in the cell cycle c. The cell cycle for an activated cell in signaling context Ω is modeled as a sequence of $G1$ phase, S phase, and $G2/M$ phase, in which the duration of latter phases (τ_S and $\tau_{G2/M}$) is fixed. In contrast to former versions of the model with fixed $G1$ phase [1, 2], this duration τ_{G1} is now randomly chosen from an exponential distribution with mean $\bar{\tau}_{G1}$ upon division of the parental cell. Cells changing into signaling context A exit from $G1$ and enter a cell cycle inactive phase ($G0$) until they are activated again and transit into signaling context Ω.
To realize an update step, the actual total number of cells with $a > a_{\min}$ in signaling context A ($N_A(t)$) and Ω ($N_\Omega(t)$) is determined. Based on these numbers, the status of each model stem cell is updated as follows:

(1) If the cell resides in signaling context A, it changes to signaling context Ω or stays in A with probabilities ω and $1 - \omega$, respectively. If it stays in A, its cell specific affinity a is increased by the factor r (regeneration coefficient), until a has reached its maximum value $a_{\max} = 1$. If the cell changes to signaling context Ω, its position in the cell cycle

will be set to the begin of S-phase.

(2) If the cell resides in signaling context Ω, it changes to context A or stays in Ω with probabilities α and $1-\alpha$, respectively. Herein, a change to signaling context A is only possible in $G1$-phase of the cell cycle for cells with $a > a_{\min}$. If the cell stays in signaling context Ω, a is decreased by the factor $1/d$ (with regeneration coefficient d) and the cell cycle position is incremented. In case of cell cycle completion (i.e., $c(t) = \tau_{G1} + \tau_S + \tau_{G2/M}$), $c(t)$ is set to 0 and a new identical cell is generated (cell division). Individual durations of $G1$ phase for both daughter cells are randomly chosen from an exponential distribution. If a has reached the minimal value $a_{\min}$, the cells cannot reenter into signaling context A and proceed development in Ω.

The transition probabilities α and ω depend on the actual affinity of the cell $a(t)$, on the fixed parameters $a_{\min}$ and $a_{\max}$, and on the transition characteristics f_α and f_ω, respectively:

$$\alpha = \frac{a(t)}{a_{\max}} f_\alpha(N_A(t)) \quad (1)$$

$$\omega = \frac{a_{\min}}{a(t)} f_\omega(N_\Omega(t)) \quad (2)$$

The transition characteristics f_α and f_ω itself, depend on the total number of cells (N_A,N_Ω) in the respective target context. They are modeled by a general class of sigmoid functions:

$$f_{\alpha/\omega}(N_{A/\Omega}) = \frac{1}{\nu_1 + \nu_2 \cdot exp\left(\nu_3 \cdot \dfrac{N_{A/\Omega}}{\tilde{N}_{A/\Omega}}\right)} + \nu_4. \quad (3)$$

The parameters ν_1, ν_2, ν_3, and ν_4 determine the shape of $f_{\alpha/\omega}$. $\tilde{N}_{A/\Omega}$ is a scaling factor for $N_{A/\Omega}$.
It is possible to uniquely determine ν_1, ν_2, ν_3, and ν_4 by the more intuitive values $f_{\alpha/\omega}(0)$, $f_{\alpha/\omega}(\frac{\tilde{N}_{A/\Omega}}{2})$, $f_{\alpha/\omega}(\tilde{N}_{A/\Omega})$, and $f_{\alpha/\omega}(\infty) := lim_{N_{A/\Omega}\to\infty} f_{\alpha/\omega}(N_{A/\Omega})$:

$$\begin{aligned} \nu_1 &= (h_1 h_3 - h_2^2)/(h_1 + h_3 - 2h_2) \\ \nu_2 &= h_1 - \nu_1 \\ \nu_3 &= \ln((h_3 - \nu_1)/\nu_2) \\ \nu_4 &= f_{\alpha/\omega}(\infty) \end{aligned}$$

with

$$\begin{aligned} h_1 &= 1\,/\,[f_{\alpha/\omega}(0) - f_{\alpha/\omega}(\infty)] \\ h_2 &= 1\,/\,\left[f_{\alpha/\omega}\left(\tilde{N}_{A/\Omega}/2\right) - f_{\alpha/\omega}(\infty)\right] \\ h_3 &= 1\,/\,[f_{\alpha/\omega}(\tilde{N}_{A/\Omega}) - f_{\alpha/\omega}(\infty)]. \end{aligned}$$

Applied parameter choices of these functions are given in Supplementary Table 1.

Within the model a representative population of HSCs includes all cells in signaling context A as well as a proportion of cells in signaling context Ω. The coverage of this HSC pool is indicated by the blue box in Figure 3 of the main document. The position of the right sided boundary of this box is defined in terms of transit time t_{trans} that a cell remains within this box after having passed the threshold value $a_{\min}$.
Motivated by the observation that even the most sophisticated protocols for the purification of HSCs yield at maximum 50 % of repopulating stem cells [3], the simulated HSC population intentionally includes a significant number of cells with restricted repopulation potential, i.e. cells $a < a_{\min}$. These cells can by definition not enter signaling context A, and are thus not able to contribute to system repopulation.

The estimated turnover times are averages over the time between two division events of an individual cell. For *activated* cells (i.e. cells are only in signaling context Ω for their whole time of existance) the individual time beween two division corresponds to the sum $\tau_{G1} + \tau_S + \tau_{G2/M}$. Cells with intermediate residence in signaling context A are denoted as *quiescent* cells. This temporary state of inactivation potentially prolongs the time between the parental division and the next division event, leading to the increased average turnover of these cells.

The mathematical model is implemented in C++ and has been tested for UNIX-derived operating systems. The source code (including parameter files for the described scenarios) can be obtained from the authors.

2 Simulation of dilution kinetics

For the simulation of label dilution kinetics each cell is additionally characterized by an individual label content $b(t)$. As the dynamics of label uptake during BrdU administration are potentially biased by the cytotoxic effects of BrdU itself [4] and because we currently have no quantitative information about this process, the model does not consider this process. Instead, upon start of label dilution a certain fraction F_0 of all HSCs receives an initial label $b(t = 0) = b_0$ (individual cells are chosen randomly within this population). Upon division the daughter cells receive half the parental label content. Cells become undetectable if the individual value $b(t)$ falls below a detection threshold b_t.

Without loss of generality we have chosen $b_0 = 0.5$ as the initial fraction of label cells and $b_0 = 0$ for the non-labeled cells. The number of divisions N that are necessary to label dilution below the level of detectibility is regulated by adjusting the detection threshold b_t.
Parameters are provided in Supplementary Table 2.

As outlined in the main document, we additionally studied the more general case in which the intial label is not fixed to $b_0 = 0.5$ but distributed according to a given probability density. Details are provided in Section 4 below.

3 Simulation of competitive retransplantations.

For the simulation of the competitive retransplantation experiments, without loss of generality, a fraction of $F_0 = 71\%$ of all HSCs is initially labeled. At different time points during label dilution, the HSC population is seperated in L+ ($b(t) > b_t$) and L- subpopulations ($b(t) < b_t$). 20 cells from each pool are retransplanted with 20 competitor cells chosen randomly from the HSC population of a corresponding model system for which the labeling routine had not been applied. Over time the 40 transplanted cells repopulate an initially empty model system and reestablish a homeostatic situation in which a fraction of cells is donor derived (originating either from the L+ or L- population) and the remaining fraction of cells originates from the pool of competitor cells. The fraction of donor derived cells represents the experimentally observable engraftment level.
Engraftment levels at each time point in Figure 5 of the main document are estimated from 100 indivdual transplantation studies. The variability of the engraftment levels (indicated by the standard deviation) results from two aspects: First, the cells are chosen randomly from the selected L+ and L- populations. Second, the engraftment is modeled as regulated, but intrisically stochastic process. Very likely, both aspects do also account for the observed variabilty in the corresponding experimental results.
To demonstrate the influence of the initial labeling routine an additional scenario is studied in which only HSCs with $a > 0.9$ are label with $b_0 = 0.5$. All other cells remain $b_0 = 0$. Due to the construction of the model, in which the cells in signaling context A accumulate at $a = 1$, the particular labeled population is almost completely composed of quiescent cells.

4 Simulation of different initial distributions of the label content b_0.

Instead of assigning the same initial label content to all cells we have additionally studied the case that b_0 is distributed around a mean value $\mu = \bar{b}_0 = 0.5$. As we have defined b in the range $[0, 1]$, we use Beta distributed random variables for this approach. The

Beta distribution with parameters $p > 0$ and $q > 0$ has density

$$f_{\text{Beta}}(x) = \frac{\Gamma(p+q)}{\Gamma(p)\Gamma(q)} x^{p-1}(1-x)^{q-1} \tag{4}$$

where Γ is the gamma function. The distribution is symmetric around $\bar{b}_0 = 0.5$ for $p = q$. Densities for different values of $p = q$ are shown in Figure 1A. Label dilution kinetics are obtained for the corresponding assignment of the initial label b_0 according to these probability densities. The detection threshold is set to $b_t = 0.02$. Results are shown in Figure 1B. Even for a highly heterogenous spread of the initial label content b_0 the dilution kinetics are closely similar to the case where all cells are initially labeled with $b_0 = 0.5$ (corresponding to $p = q = 10^6$).

References

[1] I. Roeder and M. Loeffler. A novel dynamic model of hematopoietic stem cell organization based on the concept of within-tissue plasticity. *Exp. Hematol.*, 30(8): 853–861, 2002.

[2] I. Roeder, M. Horn, I. Glauche, A. Hochhaus, M. C. Mueller, and M. Loeffler. Dynamic modeling of imatinib-treated chronic myeloid leukemia: functional insights and clinical implications. *Nat Med*, 12(10):1181–1184, 2006.

[3] Mark J Kiel, Shenghui He, Rina Ashkenazi, Sara N Gentry, Monica Teta, Jake A Kushner, Trachette L Jackson, and Sean J Morrison. Haematopoietic stem cells do not asymmetrically segregate chromosomes or retain brdu. *Nature*, 449(7159): 238–242, Sep 2007.

[4] Anne Wilson, Elisa Laurenti, Gabriela Oser, Richard C van der Wath, William Blanco-Bose, Maike Jaworski, Sandra Offner, Cyrille F Dunant, Leonid Eshkind, Ernesto Bockamp, Pietro Lio, H. Robson Macdonald, and Andreas Trumpp. Hematopoietic stem cells reversibly switch from dormancy to self-renewal during homeostasis and repair. *Cell*, 135(6):1118–1129, Dec 2008.

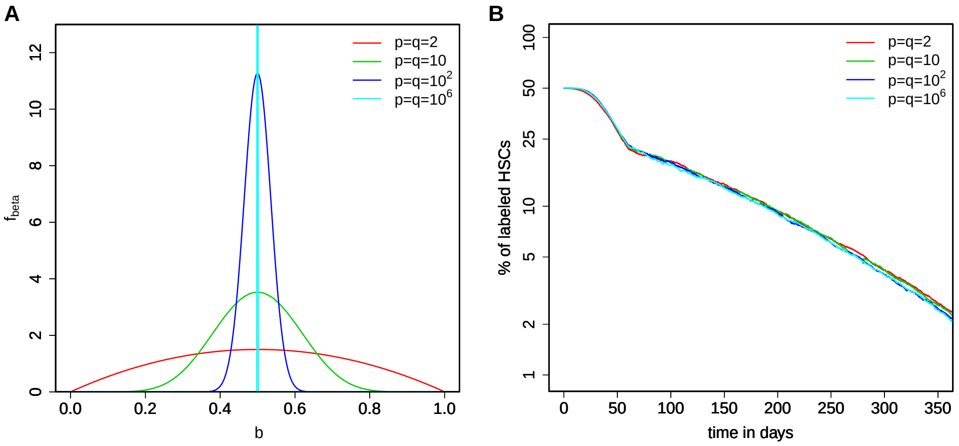

Supplementary Figure 1: **Dilution kinetics for different distributions of the initial label b_0 A.** Probability densities for different parameter configurations of the beta distribution $f_{\text{beta}}(b)$. **B.** Corresponding dilution kinetics of the label ($F_0 = 71\%$, $N = 5$ divisions until label dilution) for the different initial configurations.

parameter	value
d	1.07
r	1.1
$a_{\min}$	0.01
$a_{\max}$	1.0
$\bar{\tau}_{G1}$	480 hours
τ_S	8 hours
$\tau_{G_2/M}$	4 hours
t_{trans}	1400 hours
$f_A(0)$	0.5
$f_A(\frac{\tilde{N}}{2})$	0.3
$f_A(\tilde{N})$	0.01
$f_A(\infty)$	0
N_A^{norm}	1300
$f_\Omega(0)$	0.5
$f_\Omega(\frac{\tilde{N}}{2})$	0.0075
$f_\Omega(\tilde{N})$	0.0002
$f_\Omega(\infty)$	0
N_Ω^{norm}	280

Supplementary Table 1: **Model parameters for the simulation of the mouse model.**

parameter	simulation of the data by Kiel et al. [3] (Figure 4A)	simulation of the data by Wilson et al. [4] (Figure 4B)	general scenarios in Figures 4D	transplanations in Figure 5
F_0	45 %	71 %	71 %	71 % (*)
b_0<br>b_t	0.5<br>0.2	0.5<br>0.02	0.5<br>0.126 / 0.03126 / 0.0078126	0.5<br>0.02
N	2	5	2 / 4 / 6	5

Supplementary Table 2: **Model parameters for the different label dilution kinetics.**
(*) For Figure 5B, $F_0 = 100\%$ of the cells are labeled in a restricted region.
